# Heterogeneous effects on type 2 diabetes and cardiovascular outcomes of genetic variants and traits associated with fasting insulin

**DOI:** 10.21203/rs.3.rs-3317661/v1

**Published:** 2023-09-19

**Authors:** Alisa Manning, Magdalena Sevilla-González, Kirk Smith, Ningyuan Wang, Aubrey Jensen, Elizabeth Litkowski, Hyunkyung Kim, Daniel DiCorpo, Kenneth Westerman, Jinrui Cui, Ching-Ti Liu, Chenglong Yu, John McNeil, Paul Lacaze, Kyong-Mi Chang, Phil Tsao, Lawrence Phillips, Mark Goodarzi, Rob Sladek, Jerome Rotter, Josee Dupuis, Jose Florez, Jordi Merino, James Meigs, Jin Zhou, Sridharan Raghavan, Miriam Udler

**Affiliations:** Massachusetts General Hospital; Massachusetts General Hospital; Massachusetts General Hospital; Boston University School of Public Health; University of California; Veterans Affairs Eastern Colorado Health Care System; The Broad Institute of MIT and Harvard; Boston University School of Public Health; Massachusetts General Hospital; Cedars-Sinai Medical Center; Boston University School of Public Health; Monash University; Monash University; Monash University; The Corporal Michael J. Crescenz Veterans Affairs Medical Center and University of Pennsylvania Perelman School of Medicine; Stanford University School of Medicine; Atlanta VA Medical Center; Cedars-Sinai Medical Center; McGill University; The Lundquist Institute for Biomedical Innovation at Harbor-UCLA Medical Center; Boston University; Massachusetts General Hospital; The Broad Institute of MIT and Harvard; Department of Medicine, Harvard Medical School; University of California; University of Colorado School of Medicine

## Abstract

Hyperinsulinemia is a complex and heterogeneous phenotype that characterizes molecular alterations that precede the development of type 2 diabetes (T2D). It results from a complex combination of molecular processes, including insulin secretion and insulin sensitivity, that differ between individuals. To better understand the physiology of hyperinsulinemia and ultimately T2D, we implemented a genetic approach grouping fasting insulin (FI)-associated genetic variants based on their molecular and phenotypic similarities. We identified seven distinctive genetic clusters representing different physiologic mechanisms leading to rising FI levels, ranging from clusters of variants with effects on increased FI, but without increased risk of T2D (non-diabetogenic hyperinsulinemia), to clusters of variants that increase FI and T2D risk with demonstrated strong effects on body fat distribution, liver, lipid, and inflammatory processes (diabetogenic hyperinsulinemia). We generated cluster-specific polygenic scores in 1,104,258 individuals from five multi-ancestry cohorts to show that the clusters differed in associations with cardiometabolic traits. Among clusters characterized by non-diabetogenic hyperinsulinemia, there was both increased and decreased risk of coronary artery disease despite the non-increased risk of T2D. Similarly, the clusters characterized by diabetogenic hyperinsulinemia were associated with an increased risk of T2D, yet had differing risks of cardiovascular conditions, including coronary artery disease, myocardial infarction, and stroke. The strongest cluster-T2D associations were observed with the same direction of effect in non-Hispanic Black, Hispanic, non-Hispanic White, and non-Hispanic East Asian populations. These genetic clusters provide important insights into granular metabolic processes underlying the physiology of hyperinsulinemia, notably highlighting specific processes that decouple increasing FI levels from T2D and cardiovascular risk. Our findings suggest that increasing FI levels are not invariably associated with adverse cardiometabolic outcomes.

## Introduction

Alterations in insulin secretion and sensitivity arise many years before the development of type 2 diabetes (T2D).^1–3^ An accessible trait to characterize molecular alterations that precede the development of T2D is fasting insulin (FI), a biomarker with prognostic variability between individuals and populations.^4^ Elevated FI levels can herald defects in mechanisms of insulin secretion and sensitivity and thus inform T2D pathophysiology and coronary artery disease (CAD) risk.^5,6^ Even though genetic approaches have been helpful in detecting T2D subtypes representing distinctive pathways of deficiency of insulin production and increased insulin resistance,^7,8^ little is known about the specific molecular processes underlying FI levels and their deleterious mechanisms.

The dynamic, overlapping, and molecular complexity underlying insulin sensitivity and resistance limits our knowledge about the impact of these processes in the development of T2D.^6,9^ A better understanding of the physiological mechanisms could advance our pathophysiological understanding of T2D and its early stages, which is likely to be transformed into more targeted therapeutic and preventive strategies. While previous studies have aimed at clustering T2D genetic variants^7,8^, these approaches often provide limited insights into the molecular mechanisms underlying glycemic regulation as people with T2D often present with other metabolic alterations due to disease or treatment. To date, genome-wide association studies (GWAS) have identified hundreds of variants associated with fasting serum insulin levels ^10–14^, but very few have been functionally characterized. This is because it is clinically complex to separate functional mechanisms of insulin production and insulin action in experimental studies. Therefore, one approach to identifying molecular consequences of identified FI-associated GWAS variants are *in silico* bioinformatic analyses.

Expanding upon previous knowledge regarding the clustering of T2D loci, we undertook a genetic approach to cluster FI-related variants based on their association with body composition, inflammatory, and lipid traits in individuals without diabetes from five ancestry groups to elucidate the mechanisms that underlie the variation in insulin resistance and insulin secretion and their impact on cardiometabolic outcomes.

## Research Design and Methods

### Study design:

**Figure 1:**
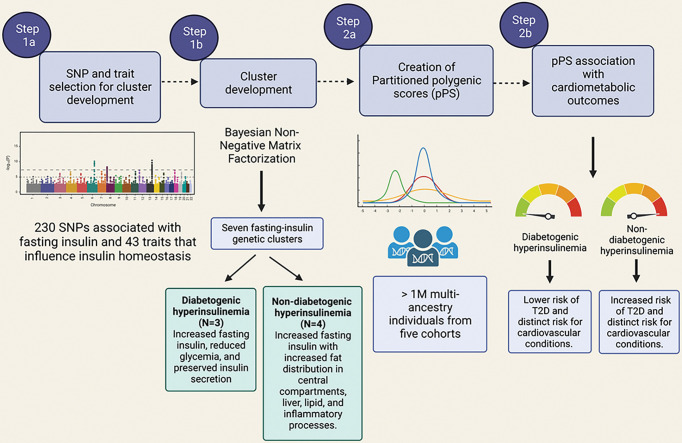
Study design and main results. This figure was created with Biorender.com.

Figure 1 summarizes our study approach and the implemented analyses. To generate FI clusters, we gathered association summary statistics for FI and related metabolic traits for FI-associated variants to identify clusters of genetic variants for increased FI sharing similar physiological and clinical characteristics. We used this information to generate partitioned polygenic scores (pPS) for the different clusters among 1,104,258 multi-ancestry individuals from five studies to investigate the association between processes underlying increased FI levels and cardiometabolic outcomes. A brief description of each contributing cohort is provided in Supplementary Table 1. Analysis of the UK Biobank was conducted under application #42614. Data analysis was approved by the Mass General Brigham Institutional Review Board (Boston, MA).

### Identification of clusters underlying increased fasting insulin levels

First, we leveraged association summary statistics to identify genetic variants previously associated with increased FI in individuals who did not have diabetes of European descent populations.^10–14^ (Supplementary Table 2). Then we obtained association summary statistics for the identified FI-associated genetic variants for traits that influence FI-levels, including association results from glycemic, anthropometric, body composition, inflammation, lipids, hormones, and liver function available in the Accelerating Medicines Partnership Common Metabolic Diseases Knowledge Portal^15^ or UK Biobank.^16^

To group genomic regions associated with FI into clusters of genetic variants with molecular and clinical similarities, we used a Bayesian non-negative matrix factorization (bNMF) clustering approach. The bNMF algorithm factorizes the inputs into two matrices with an optimal rank K, corresponding to the association matrix of variants and traits to the number of clusters.^8^ The most highly associated traits determined the main features for each cluster.^8^ The input for the bNMF approach was a set of 230 distinct genetic variants associated with FI (p < 5E-5) (Supplementary Table 3) and 43 traits with correlation |r|<0.85 between the Z-scores of each trait in the SNP-trait matrix (Supplementary Table 4). This selection of non-completely overlapping traits is critical to ensure that each phenotype provides additional information and contributes to the generation of the matrix.

The trait effect sizes were aligned to the FI risk-increasing allele, which was determined using the Lagou ^10^ (FI) and Chen (FIadjBMI) GWAS ^11^. The cut-off value to define the variants and traits that characterize each cluster was 0.84. This was determined by the optimal threshold to define the beginning of the long tail of the distribution of clusters’ weights across all clusters. The bNMF algorithm was implemented in R, and the maximum posterior solution at the most probable number of clusters was selected for downstream analysis.

### Generation of partitioned polygenic scores:

We generated pPS representing genetic susceptibility to different pathophysiological processes underlying FI levels. pPS were calculated for each study participant in each cohort by summing the weights of the gene variants derived from the bNMF clustering algorithm, as has been described before (Supplementary Table 5–6).^7,8,17^ To generate pPS, we used directly genotyped variants or imputed variants. If a genetic variant was unavailable or ambiguous, we used available proxies based on r^2^ ≥ 0.8. To prevent unintentional allele swapping, the frequencies of the alleles used in developing the clusters for hyperinsulinemia were compared with European ancestry individuals from the Mass General Brigham Biobank (MGBB). The genotyping information and imputation methods from the different studies included in our study are described in Supplementary Table 1. The median and distribution of the pPS were comparable across included studies.

### Outcome ascertainment:

The primary outcomes were T2D, CAD, myocardial infarction (MI), stroke, ischemic stroke (ISTR), hypertension (HTN), chronic kidney disease (CKD), and estimated glomerular filtration rate (eGFR). The rationale for investigating these outcomes was because of the association of FI levels with metabolic, renal, and cardiovascular conditions. Outcome definitions were generally consistent across studies, and the study-specific definitions can be found in Supplementary Table 1.

Secondary outcomes included diabetes complications defined as neuropathy, retinopathy, kidney disease occurring in individuals with diabetes, and insulin use. Outcome definitions can also be found in Supplementary Table 1.

### Association between partitioned polygenic scores and cardiometabolic outcomes:

We elaborated and circulated a harmonized statistical analysis plan with a standardized definition of pPS, statistical models, covariates, and outcome definitions. In each cohort, we used multivariable regression models to estimate effect sizes and the odds of primary outcomes for each pPS. We computed pPS on a continuous scale and presented estimates for 10 unit increase in polygenic scores. Models were adjusted for age, sex, and the 10 principal components (PCs) of genetic ancestry. For analyses conducted in UK Biobank, only PCs that reached the nominal significance threshold for association in the null model were included. In a secondary analysis, in all cohorts, we categorized individuals according to the distribution of pPS and conducted association analyses comparing individuals in the top 10th distribution of each pPS against the rest. Because the median and distribution of the polygenic scores across the cohorts were consistent, we used the MGBB cohort to standardize the cutoff point to define high genetic risk among the five cohorts.

For multi-ancestry cohorts, we conducted subgroup analyses stratified by race/ethnicity. To ensure accurate race/ethnic classification of participants, we employed a combination of self-reported race/ethnicity and genetic-derived continental ancestry approaches. We first classified individuals with their self-reported race/ethnicity and then cross-referenced this information with their genetic PCs to verify the accuracy of the classification. Individuals who did not match their self-reported race/ethnicity with their genetic PCs were excluded from the specific ancestry group. The MVP study used the Harmonized Ancestry and Race Ethnicity (HARE) algorithm^18^. We also conducted subgroup analyses stratified by T2D status.

Outcome-specific study-level regression coefficients were combined by inverse-variance weighted fixed effects meta-analysis. The R package “meta” version 4.18–2 was used to combine estimates of effect and produce an overall association test. We used the *I*^*2*^ statistic to assess between-study heterogeneity. We considered a two-sided α level of 0.0008 based on a Bonferroni adjustment for performing 56 tests, including eight outcomes and seven genetic clusters. Statistical analyses were carried out using R software, versions 3.5.1 and 4.1.1.

## Results

**Figure 2:**
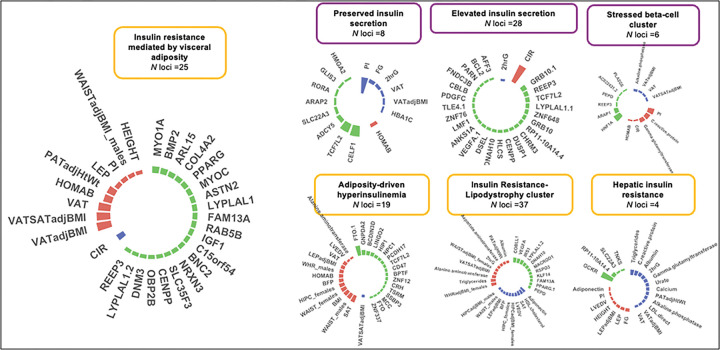
Seven potential mechanisms for hyperinsulinemia; Each cluster is represented by a set of loci and a set of traits; The loci and traits represented in the plots are the top-weighted for better visualization; clusters might have more variants and traits. Loci with a number after the period “. xx” represents that there was more than one SNP at that locus. The bars represent the weights with which they contribute to each cluster; In the case of traits, the color of the bars represents the direction of the effect that was found in each cluster, red for the positive association and blue for the negative association. VAT, Visceral Adipose Tissue; SAT, Subcutaneous Adipose Tissue; 2hrG, Glucose at 2 hours after an Oral Glucose Tolerance Test; LEP, Leptin; HDL, High-density Lipoprotein; LVEDV, Left Ventricular end-diastolic volume; WHR, Waist hip ratio; PATadjHtWt: Pericardial Adipose Tissue adjusted by Hight to Weight ratio; HOMAB, Homeostasis Model Assessment of β-cell function; HIPC, Hip circumference; BFP, Body Fat percentage; VATSAT, VAT:SAT ratio; PI, Proinsulin; CIR, Corrected Insulin Response; FG, Fasting Glucose; HBA1C, Hemoglobin A1C; LDL, Low-density Lipoprotein. Purple lines distinguished non-diabetogenic hyperinsulinemia clusters; increased fasting insulin, reduced glycemia, and preserved insulin secretion. Yellow lines distinguish clusters of diabetogenic hyperinsulinemia; increased fasting insulin due to a strong effect on body fat distribution, liver, lipid, and inflammatory processes.

Using extant association summary statistics, we identified seven distinctive genetic clusters with defined genetic and trait similarities. The loci and traits defining each cluster are described in Supplementary Table 5 and Fig. 2. We classified the seven genetic clusters based on their association with T2D (which was not an input in the clustering). While all genetic clusters were defined by alleles increasing FI levels, three clusters were associated with non-elevated T2D risk (non-diabetogenic) and four with increased T2D risk as well as strong effects on body fat distribution, liver, lipid, and inflammatory processes (diabetogenic hyperinsulinemia).

Among the three clusters characterized by non-diabetogenic hyperinsulinemia, the first is a subset of genomic regions associated with increasing FI with concomitant effects on lower glycemic traits (fasting glucose, 2h glucose, or A1c). Genetic variants in this cluster denoted processes underlying increased FI levels with preserved beta-cell function, and *CELF1* and *TCF7L2* were among the eight top-weighted loci, which we refer to as “preserved insulin secretion” cluster. The second non-diabetogenic hyperinsulinemia cluster was a subgroup of increasing FI genetic variants with overlapping effects on increased corrected insulin response and decreased 2h glucose, denoting a putative pathway of increased insulin secretion (referred to as “elevated insulin secretion cluster”), this cluster has *GRB10* and *REEP3* as the 28 top-weighted loci. A third cluster of genetic variants associated with increased FI levels with subtle effects on glucose homeostasis involves a subset of genetic variants associated with increased levels of proinsulin, C-reactive protein, and gamma glutamine transferase (GGT) with *HNF1A* and *ARAP1* among the six top-weighted loci. This cluster recapitulates a beta cell under stress, which we call “stressed beta-cell cluster” (Fig. 2).

We also identified four different mechanisms underlying impaired insulin sensitivity and increased risk of T2D, or diabetogenic hyperinsulinemia. First, we identified a subgroup of variants in which the FI-increasing alleles were associated with increased waist circumference, body fat percentage, and subcutaneous adipose tissue (SAT). This suggests evidence of a cluster characterized by increased FI driven by generalized adiposity with *FTO* among the top-weighted 19 loci. Second, we identified a visceral adiposity-mediated insulin resistance cluster with a strong confluence of genetic variants for increased FI with increased visceral adipose tissue (VAT), visceral to subcutaneous adipose tissue ratio, and lower corrected insulin response. In this cluster, the 25 top-weighted loci included the *MYO1A, BMP2*, and *ARL15* genes. Third, a distinct subset was characterized by increasing FI genetic variants concomitantly associated with increased fat distribution in central compartments and a detrimental circulating and hepatic lipid deposition, which we called the “insulin resistance-lipodystrophy cluster”. The top 37 highly weighted loci in included *PPARG, IRS1, LYPLAL1*, and *DNAH10* genes. Finally, we identified a subgroup of genetic variants with a concomitant effect on insulin sensitivity and alterations in liver metabolism and inflammation, which could correspond to hepatic insulin-resistance processes, which we called the “hepatic-insulin resistance cluster”. FI-increasing alleles converging in this cluster are associated with lower circulating triglycerides, albumin, and C-reactive protein, and *GCKR* was among the four top-weighted loci. (Fig. 2).

### Associations between partitioned polygenic scores, T2D, and cardiometabolic outcomes

We constructed pPS denoting genetic susceptibility to these seven distinct mechanisms underlying FI levels among 1,104,258 individuals from five studies. The pPS showed similar distributions across cohorts and populations (Supplementary Table 7).

**Figure 3:**
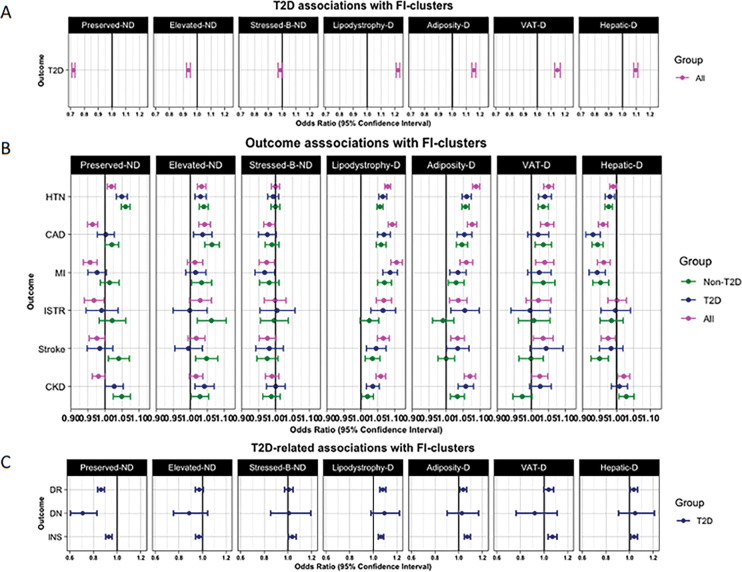
The plot shows meta-analysis results from five cohorts UKBB, MVP, ASPREE, MGBB, and FHS. Subgroup results are differentiated by colors, green (non-T2D), blue (T2D). IR: Insulin resistance, IS insulin secretion. Panel A. shows T2D, type 2 diabetes associations with fasting insulin genetic clusters. Panel B shows associations of fasting insulin clusters with cardiometabolic outcomes; HTN, Hypertension; CAD, Coronary artery disease; MI, Myocardial Infarction; ISTR, Ischemic Stroke; CKD, Chronic Kidney Disease. Panel C shows associations of fasting insulin genetic clusters with T2D-exclusive outcomes; DR, Diabetic Retinopathy; Diabetic Neuropathy; INS, insulin use. Fasting-insulin genetic lusters show distinctive patterns of association with cardiometabolic outcomes, ranging from protective mechanisms to high-risk associations.

Our combined analyses showed that the clusters defined by non-diabetogenic hyperinsulinemia were associated with non-increased risks of T2D, yet differing risks of CAD (Fig. 3 Supplementary Table 8). For example, in all individuals (with and without T2D), each 10-unit increase in the pPS of the genetic cluster characterized by preserved insulin secretion was associated with lower odds of T2D (OR 0.72, *P* < 10^−300^), CAD (OR 0.96, *P* < 10^−6^), MI (OR 0.95, *P* < 10^−5^). The lower T2D odds was also observed for the elevated insulin secretion cluster (OR 0.93, *P* < 10^−16^), but this cluster, unlike the previous one, was associated with increased odds of CAD (OR 1.04, *P* < 10^−7^), and HTN (OR 1.03, *P* < 10^−8^). No associations with metabolic outcomes were observed for the stressed beta-cell cluster.

The pPS for the four genetic clusters underlying processes of diabetogenic hyperinsulinemia were all generally associated with an increased risk of T2D, with estimated effect sizes ranging from 1.22 (*P* < 10^−300^) for the insulin resistance-lipodystrophy cluster to 1.09 (*P* < 10^−40^) for the hepatic insulin-resistance cluster (Fig. 3, Supplementary Table 8). However, directions of associations differed for metabolic outcomes, including CAD, MI, stroke, and eGFR. For example, the insulin resistance-lipodystrophy cluster was also associated with lower eGFR, increased odds for CKD, and most cardiovascular outcomes considered in this study, including CAD, MI, and stroke, while the hepatic insulin resistance cluster was associated with lower odds for CAD, MI, and lower eGFR (Fig. 3).

Further, we investigated the extent to which these associations were consistent when individuals were categorized as having extreme genetic risk (> 90th percentile of a pPRS) for these processes. These analyses were overall consistent with our primary results (Supplementary Table 9), supporting the notion that the mechanisms by which these processes cause hyperinsulinemia have different impacts on cardiometabolic conditions.

In ancestry-specific analyses (Supplementary Table 10), we found that the associations between FI-pPS and T2D susceptibility had the same direction of effect among Hispanic individuals, although with considerable differences in effect sizes compared to European ancestry individuals for five out of the seven genetic clusters. Analyses restricted to non-Hispanic Black participants had the same direction of effect as European-ancestry individuals for three of the cluster pPS: Preserved Insulin Secretion Cluster, Insulin Resistance-Lipodystrophy Cluster, and Elevated Insulin Secretion Cluster. Only two out of the seven FI-pPS associations, Preserved Insulin Secretion Cluster, and Insulin Resistance-Lipodystrophy Cluster were replicated among South Asian ancestry individuals, and none was observed in East Asian ancestry participants.

In a subgroup analysis to evaluate complications in individuals with T2D (n = 208,268), we observed that the preserved insulin secretion cluster was associated with lower odds of diabetic neuropathy (OR, 0.70, *P* < 10^−5^) diabetic retinopathy (OR 0.86 *P* < 10^−18^), and insulin use (OR 0.93, *P* < 10^−7^) (Fig. 3, Supplementary Table 8). These results expanded our previous observation showing that the preserved insulin secretion cluster was associated with lower odds of T2D. Among the genetic clusters defined by elevated FI levels with deeper metabolic alterations, we found that insulin resistance-lipodystrophy and adiposity-mediated insulin resistance genetic clusters exhibited the largest number of associations with T2D complications (Fig. 3, Supplementary Table 8) including MI, diabetic retinopathy, CKD, ISTR, and insulin use. Interestingly in individuals without T2D, (n = 895,990), we observed that the lipodystrophy (*P* < 10^−17^), adiposity (*P* < 10^−8^), and elevated insulin secretion (*P* < 10^−9^) genetic clusters maintained their associations with increased CAD and MI risk (OR 1.06, *P* < 10^−12^) (Fig. 3, Supplementary Table 8).

## Discussion

We identified seven distinctive genetic clusters representing different molecular processes for FI levels, ranging from clusters of variants with effects on increased FI, and non-increased glycemia (non-diabetogenic hyperinsulinemia), to clusters of variants that increased FI and T2D risk with demonstrated strong effects on body fat distribution, liver, lipid, and inflammatory processes (diabetogenic hyperinsulinemia). In addition, our findings provide important insights into granular metabolic processes that decouple increasing FI levels from cardiovascular conditions. Clusters characterized by non-diabetogenic hyperinsulinemia were associated with non-increased risks of T2D, and both increased and decreased risks of cardiovascular conditions. In contrast, diabetogenic hyperinsulinemia clusters were associated with an increased risk of T2D and at the same time different risks for cardiovascular conditions, including CAD, MI, and stroke.

This work expands upon previous studies from Udler et al ^7,8^. regarding the clustering of T2D loci, adding a more granular analysis of the mechanisms underlying T2D and cardiometabolic risk. Our results support the notion that insulin resistance associated with increased fat distribution in central compartments is a key contributing factor. Both the traits and genetic loci comprising the diabetogenic hyperinsulinemia clusters reflect this adverse metabolic physiology. The top loci in the insulin resistance mediated by visceral adiposity cluster *MYO1A, BMP2*, and *ARL5* have been previously described for their association with lower BMI and their role in the partitioning of energy storage into visceral and subcutaneous adipose tissue depots^19,20^. The insulin resistance mediated by reduced subcutaneous adipose tissue cluster relates to a “lipodystrophy-like” phenotype previously described by Yaghootkar ^21^, Lotta^22^ and Udler ^7^. The weighted loci in this cluster (*N* = 37 loci) are lipodystrophy and adiposity-related variants (*PPARG, IRS1, LYPLAL1*, and *DNAH10*). Some of these variants lie in or near genes implicated in monogenic forms of lipodystrophy. Moreover, the insulin resistance mediated by the adiposity cluster appears to represent a mechanism of insulin resistance concomitant with increments in body fat. Top-weighted loci are well known for their association with BMI (*FTO, GNPDA2*, and *BCDIN3D*)^23–25^. In addition, our findings suggest that excessive visceral adiposity and ectopic fat strongly contribute to cardiovascular risk ^26^. Consistent with an adverse impact of excessive visceral adiposity and ectopic fat, the diabetogenic hyperinsulinemia clusters accurately captured the heightened risk of cardiovascular outcomes such as HTN, CAD, MI, ISTR, stroke, and CKD, associations that remained consistent regardless of the presence of T2D.

The non-diabetogenic hyperinsulinemia clusters associated with non-increased T2D risk exhibited characteristics indicative of preserved insulin production. These mechanisms in the preserved beta-insulin secretion cluster can partially be mediated by ADCY5 which has been implicated in the regulation of insulin secretion from human islets ^27^. This cluster appears to capture the opposite alleles as those in the T2D beta-cell cluster described by Udler et al.^7^ A possible explanation is that in our study, the effect allele in *ADCY5* has been associated with higher expression levels and higher levels of insulin secretion and reduced T2D risk compared to the reference allele found in Udler et al^7^. The increments in insulin production might be interpreted as variants that enhance or preserve beta-cell function (*ADCY5*) potentially leading to lower blood glucose levels and thus lower cardiometabolic risk or it can also be explained by an early compensatory mechanism when glycemia is normal or slightly elevated ^2,28,29^.

We identified two clusters where the association with T2D was decoupled from CAD risk, one diabetogenic and one non-diabetogenic cluster. The hepatic-insulin resistance cluster was the only diabetogenic cluster that exhibited evidence of decreased cardiovascular risk. Top weighted loci in this cluster have been connected to fatty liver disease (*GCKR*)^30^ and hepatic glycogen storage (*RP11-10A14.4/PPP1R3B*) ^31–33^, and the top-weighted trait was decreased serum triglyceride levels, suggesting that the cluster association with reduced CAD risk might be explained by loci impacting hepatic storage of triglycerides and/or glycogen. This cluster correlates with the liver lipid cluster previously identified by Udler *et al*.^7^, and is consistent with the finding by Di Corpo et. al^17^ that adjusting for serum triglycerides in a regression model partially attenuated the associations with CAD.

The second instance of decoupled associations between T2D and CAD involved a non-diabetogenic cluster that was characterized by elevated insulin secretion. In individual-level data analyses, this cluster was significantly associated with reduced T2D risk but increased CAD risk. The underlying mechanisms related to these associations remain unclear and warrant further investigation.

This study exhibits several strengths; first, the large-scale GWAS provided the necessary data to establish FI clusters for various pertinent outcomes. It utilized a substantial dataset (comprising over a million individuals) from diverse ancestries to assess both the correlation between these clusters and prevalent disease states and their ability to distinguish between diabetes and non-diabetes. The study also has some limitations; we acknowledge some overlap in the GWAS discovery studies and the multi-ancestry cohort outcome studies. This inclusion may introduce overfitting in our models; however, we assessed the meta-analysis association analysis with and without the FHS cohort and did not observe significant differences in our results. Another limitation is that SNPs and traits GWAS summary statistics utilized in the bNMF algorithm represent studies conducted in individuals of European ancestry only. To further enhance our understanding of the behavior of these clusters across diverse populations, future research should encompass multi-ancestry GWAS results.

Our clustering approach offers valuable insights to decouple hyperinsulinemia from T2D and cardiovascular conditions, presenting a novel avenue for studying precision medicine strategies. The outcomes of this study can aid in identifying groups that require targeted therapeutic approaches, or exploring pathways aimed at preventing disease onset. This represents a significant stride towards genetically informed patient prevention and management of T2D, which is crucial given the limitations of current therapeutic approaches in addressing disease heterogeneity and predicting future disease trajectories.

## Conclusion

Through the examination of a key intermediate trait, FI, we gained detailed insights into the heterogeneity of T2D and cardiovascular conditions. Our findings challenge the assumption that increasing FI levels always correlates with unfavorable outcomes. By clustering FI-related loci and traits, we identified seven genetic clusters of FI that have both increasing and decreasing effects on CAD risk which are not always aligned with T2D risk. Further research is required to investigate the potential clinical and therapeutic implications of these distinct clusters.

## Data Availability

The UK Biobank (UKB) whole-genome sequence data can be accessed through UKB Research Analysis Platform (RAP), through the UKB approval system (https://www.ukbiobank.ac.uk). The Mass General Brigham Biobank (MGBB) individual-level data are available from https://personalizedmedicine.partners.org/Biobank/Default.aspx, where the data is available through institutional review board (IRB) approval and therefore not publicly available. ASPirin in Reducing Events in the Elderly Study data is available through internal approval https://ams.aspree.org/public/ The Framingham Heart Study genomic data analyzed in the current study are available through restricted access via the Genotypes and Phenotypes (dbGaP) Exchange area in the database of repositories phs000974 and phs000007. The Million Veterans Program data that support the findings of this study are not openly available due to reasons of sensitivity and are available from the corresponding author upon reasonable request.

## References

[1] Taylor R. Insulin Resistance and Type 2 Diabetes. Diabetes 61, 778 (2012). 22442298 10.2337/db12-0073 PMC3314346

[2] Stumvoll M., Goldstein B. J. & Van Haeften T. W. Type 2 diabetes: principles of pathogenesis and therapy. Lancet 365, 1333–1346 (2005). 15823385 10.1016/S0140-6736(05)61032-X

[3] Samuel V. T. & Shulman G. I. The pathogenesis of insulin resistance: integrating signaling pathways and substrate flux. J Clin Invest 126, 12–22 (2016). 26727229 10.1172/JCI77812 PMC4701542

[4] MO G. Fasting insulin reflects heterogeneous physiological processes: role of insulin clearance. Am J Physiol Endocrinol Metab 301, (2011). 10.1152/ajpendo.00013.2011 PMC3154529 21632466

[5] Després J.-P. Hyperinsulinemia as an independent risk factor for ischemic heart disease. N Engl J Med 334, 952–958 (1996). 8596596 10.1056/NEJM199604113341504

[6] Wang F., Han L. & Hu D. Fasting insulin, insulin resistance and risk of hypertension in the general population: A meta-analysis. Clin Chim Acta 464, 57–63 (2017). 27836689 10.1016/j.cca.2016.11.009

[7] Udler M. S. Type 2 diabetes genetic loci informed by multi-trait associations point to disease mechanisms and subtypes: A soft clustering analysis. PLoS Med 15, (2018). 10.1371/journal.pmed.1002654 PMC6150463 30240442

[8] Kim H. High-throughput genetic clustering of type 2 diabetes loci reveals heterogeneous mechanistic pathways of metabolic disease. Diabetologia 2022 66:3 66, 495–507 (2022). 10.1007/s00125-022-05848-6 PMC10108373 36538063

[9] Gutch M., Kumar S., Razi S. M., Gupta K. & Gupta A. Assessment of insulin sensitivity/resistance. Indian J Endocrinol Metab 19, 160 (2015). 25593845 10.4103/2230-8210.146874 PMC4287763

[10] Lagou V. Sex-dimorphic genetic effects and novel loci for fasting glucose and insulin variability. Nat Commun 12, (2021). 10.1038/s41467-020-19366-9 PMC7785747 33402679

[11] Chen J. The Trans-Ancestral Genomic Architecture of Glycemic Traits. Nat Genet 53, 840 (2021). 34059833 10.1038/s41588-021-00852-9 PMC7610958

[12] Laakso M. The Metabolic Syndrome in Men study: a resource for studies of metabolic and cardiovascular diseases. J Lipid Res 58, 481–493 (2017). 28119442 10.1194/jlr.O072629 PMC5335588

[13] Locke A. E. Exome sequencing of Finnish isolates enhances rare-variant association power. Nature 572, 323 (2019). 31367044 10.1038/s41586-019-1457-z PMC6697530

[14] Dupuis J. New genetic loci implicated in fasting glucose homeostasis and their impact on type 2 diabetes risk. Nat Genet 42, 105 (2010). 20081858 10.1038/ng.520 PMC3018764

[15] Common Metabolic Disease Knowledge Portal - Home. https://hugeamp.org/.

[16] UK Biobank — Neale lab. http://www.nealelab.is/uk-biobank.

[17] DiCorpo D. Type 2 Diabetes Partitioned Polygenic Scores Associate With Disease Outcomes in 454,193 Individuals Across 13 Cohorts. Diabetes Care 45, 674–683 (2022). 35085396 10.2337/dc21-1395 PMC8918228

[18] Fang H. Harmonizing Genetic Ancestry and Self-identified Race/Ethnicity in Genome-wide Association Studies. Am J Hum Genet 105, 763–772 (2019). 31564439 10.1016/j.ajhg.2019.08.012 PMC6817526

[19] Klimentidis Y. C. & Arora A. Interaction of Insulin Resistance and Related Genetic Variants With Triglyceride-Associated Genetic Variants. Circ Cardiovasc Genet 9, 154–161 (2016). 26850992 10.1161/CIRCGENETICS.115.001246 PMC4838530

[20] Guiu-Jurado E. Bone morphogenetic protein 2 (BMP2) may contribute to partition of energy storage into visceral and subcutaneous fat depots. Obesity (Silver Spring) 24, 2092–2100 (2016). 27515773 10.1002/oby.21571

[21] Yaghootkar H. Genetic evidence for a normal-weight ‘metabolically obese’ phenotype linking insulin resistance, hypertension, coronary artery disease, and type 2 diabetes. Diabetes 63, 4369–4377 (2014). 25048195 10.2337/db14-0318 PMC4392920

[22] Lotta L. A. Integrative genomic analysis implicates limited peripheral adipose storage capacity in the pathogenesis of human insulin resistance. Nature Genetics 2016 49:1 49, 17–26 (2016). 10.1038/ng.3714 PMC5774584 27841877

[23] Peng S. FTO gene polymorphisms and obesity risk: A meta-analysis. BMC Med 9, 1–15 (2011). 21651756 10.1186/1741-7015-9-71 PMC3118373

[24] Cheung C. Y. Y. Genetic variants associated with persistent central obesity and the metabolic syndrome in a 12-year longitudinal study. Eur J Endocrinol 164, 381–388 (2011). 21147891 10.1530/EJE-10-0902

[25] Tang L. Meta-analyses between 18 candidate genetic markers and overweight/obesity. Diagn Pathol 9, 1–12 (2014). 24621099 10.1186/1746-1596-9-56 PMC4008255

[26] Després J. P. Body Fat Distribution and Risk of Cardiovascular Disease. Circulation 126, 1301–1313 (2012). 22949540 10.1161/CIRCULATIONAHA.111.067264

[27] Hodson D. J. ADCY5 Couples Glucose to Insulin Secretion in Human Islets. Diabetes 63, 3009–3021 (2014). 24740569 10.2337/db13-1607 PMC4141364

[28] Efrat S. Beta-cell expansion for therapeutic compensation of insulin resistance in type 2 diabetes. Int J Exp Diabesity Res 4, 1–5 (2003). 12745664 10.1080/15438600303731 PMC2480502

[29] Cerf M. E. Beta cell dynamics: beta cell replenishment, beta cell compensation and diabetes. Endocrine 44, 303–311 (2013). 23483434 10.1007/s12020-013-9917-y

[30] Li J. Contribution of Rs780094 and Rs1260326 Polymorphisms in GCKR Gene to Non-alcoholic Fatty Liver Disease: A Meta-Analysis Involving 26,552 Participants. Endocr Metab Immune Disord Drug Targets 21, 1696–1708 (2020). 10.2174/1871530320999201126202706 33243135

[31] Kahali B. A Noncoding Variant near PPP1R3B Promotes Liver Glycogen Storage and MetS, but Protects against Myocardial Infarction. Journal of Clinical Endocrinology and Metabolism 106, 372–387 (2021). 33231259 10.1210/clinem/dgaa855 PMC7823249

[32] Stender S. Relationship between genetic variation at PPP1R3B and levels of liver glycogen and triglyceride. Hepatology 67, 2182–2195 (2018). 29266543 10.1002/hep.29751 PMC5991995

[33] Manning A. K. A Long Non-coding RNA, LOC157273, Is an Effector Transcript at the Chromosome 8p23.1-PPP1R3B Metabolic Traits and Type 2 Diabetes Risk Locus. Front Genet 11, 521982 (2020). 10.3389/fgene.2020.00615 PMC7367044 32754192

